# Long Noncoding RNA RP11-278A23.1, a Potential Modulator of p53 Tumor Suppression, Contributes to Colorectal Cancer Progression

**DOI:** 10.3390/cancers16050882

**Published:** 2024-02-22

**Authors:** Masayo Kamikokura, Shoichiro Tange, Hiroshi Nakase, Takashi Tokino, Masashi Idogawa

**Affiliations:** 1 Department of Medical Genome Sciences, Cancer Research Institute, Sapporo Medical University School of Medicine, Sapporo 060-8556, Japan; k-masayo@sapmed.ac.jp (M.K.); stange@sapmed.ac.jp (S.T.); tokino@sapmed.ac.jp (T.T.); 2 Department of Gastroenterology and Hepatology, Sapporo Medical University School of Medicine, Sapporo 060-8556, Japan; hiro_nakase@sapmed.ac.jp

**Keywords:** lncRNA, p53, colorectal cancer, apoptosis

## Abstract

**Simple Summary:**

To identify lncRNAs contributing to colorectal cancers, we screened lncRNAs through expression and survival analyses in datasets from The Cancer Genome Atlas (TCGA). The screen revealed that RP11-278A23.1 expression is significantly increased in colorectal cancer tissues compared with normal tissues and that high RP11-278A23.1 expression correlates with poor prognosis. The knockdown of RP11-278A23.1 inhibited the growth of and promoted apoptosis in colorectal cancer cells. Interestingly, the RP11-278A23.1 knockdown altered the expression of several proapoptotic genes in colorectal cancer cells not only with wild-type p53 but also with mutated p53. These results suggest that RP11-278A23.1 modifies the expression of these apoptosis-related genes in p53-dependent and p53-independent manners. In summary, lncRNA RP11-278A23.1 contributes to colorectal cancer progression by promoting cell growth and inhibiting apoptosis, suggesting that this lncRNA may be a useful therapeutic target.

**Abstract:**

Recently, many studies revealed that long noncoding RNAs (lncRNAs) play important roles in cancers. To identify lncRNAs contributing to colorectal cancers, we screened lncRNAs through expression and survival analyses in datasets from The Cancer Genome Atlas (TCGA). The screen revealed that RP11-278A23.1 expression is significantly increased in colorectal cancer tissues compared with normal tissues and that high RP11-278A23.1 expression correlates with poor prognosis. The knockdown of RP11-278A23.1 inhibited the growth of and promoted apoptosis in colorectal cancer cells. Next, to comprehensively examine differentially expressed genes after RP11-278A23.1 knockdown, RNA sequencing was performed in HCT116 cells. The expression of p21, a p53 target gene, was significantly upregulated, and the expression of several p53 target proapoptotic genes was also altered. RP11-278A23.1 knockdown increased p53 expression at the translational level but not at the transcriptional level. Interestingly, RP11-278A23.1 knockdown also altered the expression of these proapoptotic genes in DLD1 cells with mutated p53 and in p53-knockout HCT116 cells. These results suggest that RP11-278A23.1 modifies the expression of these apoptosis-related genes in p53-dependent and p53-independent manners. In summary, lncRNA RP11-278A23.1 contributes to colorectal cancer progression by promoting cell growth and inhibiting apoptosis, suggesting that this lncRNA may be a useful therapeutic target.

## 1. Introduction

With the completion of the Human Genome Project in 2003 and the decoding of the human genome, it was found that protein-coding regions account for approximately 2% of the entire genome [1], and most functions of intergenic regions remained unclear. However, advances in transcriptome analysis revealed that many noncoding RNAs are transcribed from such regions [2]. Noncoding RNAs exist in a variety of types and lengths and include ribosomal RNAs (rRNAs), transfer RNAs (tRNA), and small noncoding RNAs of 200 or fewer bases, such as small nuclear RNAs (snRNAs), small nucleolar RNAs (snoRNAs), microRNAs (miRNAs), small interfering RNAs (siRNAs), piwi-interacting RNAs (piRNAs), and long noncoding RNAs (lncRNAs) of 200 or more bases [3]. Among these types of RNAs, lncRNAs were shown to mechanistically regulate gene expression via their various functions, such as guide, scaffold, decoy, miRNA sponge, miRNA precursor, and chromatin looping functions, and are involved in various biological and pathological processes, such as development, differentiation, stemness, and carcinogenesis [4]. In a previous study, we found that the lncRNA NEAT1 is a direct transcriptional target of p53, and the knockdown of NEAT1 affects p53 transcriptional activation and promotes cell growth. Furthermore, the low expression of NEAT1 correlates with poor prognosis in colorectal, lung, and breast cancers [5]. However, the functions of only a few lncRNAs were clarified because there are myriad lncRNAs.

Colorectal cancer has high incidence and mortality rates worldwide [6]. Several studies reported that certain lncRNAs contribute to the progression of colorectal cancer, such as HOTAIR, MALAT-1, and CCAT1/CCAT2 [7,8,9,10]. HOTAIR expression is increased in colorectal cancers and is associated with metastasis and poor prognosis [11,12,13,14,15]. Similarly, MALAT-1 is also upregulated in colorectal cancers and promotes the proliferation of colorectal cancer cells [16,17,18]. CCAT1 and CCAT2 are located in 8q24.21 and are frequently amplified in colorectal cancers, and their high expression of CCAT1 and CCAT2 is associated with poor prognosis [19,20,21,22]. 

In this study, we screened lncRNAs through expression and survival analyses in datasets from The Cancer Genome Atlas (TCGA) to identify novel lncRNAs that contribute to the progression of colorectal cancers.

## 2. Materials and Methods

### 2.1. Analysis of Gene Expression Datasets

RNA-seq bam files of colorectal cancers (TCGA-COAD) were downloaded from the Genomic Data Commons (GDC) portal site (https://portal.gdc.cancer.gov, accessed on 30 July 2018), and gene expression was quantified using Cufflinks software v2.2.1. Patients represented in the datasets were divided into two groups: a high RP11-278A23.1 expression group and a low RP11-278A23.1 expression for best discrimination. A survival curve was constructed via the Kaplan–Meier method using survfit (R package: survival v3.5.5). *p* values were calculated with the log-rank test using survdiff (R package: survival v3.5.5) as previously described [23].

### 2.2. Cell Culture

The human colon cancer HCT116 and DLD1 cells were obtained from the American Type Culture Collection and the Japan Collection of Research Bioresources. The colon cancer line HCT116 (p53+/+) cells and its derivative HCT116 (p53−/−) cell line were generously provided by Dr. Bert Vogelstein (Howard Hughes Medical Institute, Johns Hopkins University, Baltimore, MD, USA). These cells were authenticated by a short tandem repeat (STR) analysis service (BEX, Tokyo, Japan) and then maintained in our laboratory. These cells were confirmed to be free of mycoplasma contamination with a Mycoplasma Detection Kit (Takara Bio, Kusatsu, Japan).

### 2.3. siRNAs and Expression Vectors

si-RP11-278A23.1 #1 (sense, 5′-CUGUAUAACUGGGAAUACA[dT][dT]-3′; antisense, 5′-UGUAUUCCCAGUUAUACAG[dT][dT]-3′), si-RP11-278A23.1 #2 (sense, 5′-CAAGUCUGGCCUCUGGUCA[dT][dT]-3′; antisense, 5′-UGACCAGAGGCCAGACUUG[dT][dT]-3′), si-KLHDC4 #1 (sense, 5′-GAGCUCUAUGUCUACAAUA[dT][dT]-3′; antisense, 5′-UAUUGUAGACAUAGAGCUC[dT][dT]-3′), si-KLHDC4 #2 (sense, 5′-CGAAUCACCAGACACUGUU[dT][dT]-3′; antisense, 5′-AACAGUGUCUGGUGAUUCG[dT][dT]-3′), and the control siRNA (siRNA Universal Negative Control #1) were purchased from Merck (Darmstadt, Germany). Transfection of siRNAs was performed using Lipofectamine RNAiMAX reagent (Thermo Fisher Scientific, Waltham, MA, USA) according to the manufacturer’s instructions. Plasmids for the expression of RP11-278A23.1, Kelch Domain Containing 4 (KLHDC4), KLHDC4 transcript variant X7, and EGFP (control) were constructed using pRP[Exp]-Puro-CAG by Vector Builder. Transfection of plasmids was performed using Lipofectamine 2000 reagent (Thermo Fisher Scientific) according to the manufacturer’s instructions.

### 2.4. RNA Extraction and Reverse Transcription–Quantitative PCR (RT–qPCR)

Total RNA was prepared from the cell lines using an RNeasy Mini Kit (QIAGEN, Hilden, Germany). For RT–PCR, cDNA was synthesized from 5 μg of total RNA with ReverTra Ace qPCR RT Master Mix with gDNA Remover (TOYOBO, Osaka, Japan). TaqMan Fast Advanced Master Mix and a QuantStudio3 Real-time PCR system (Thermo Fisher Scientific) were used for qPCR according to the manufacturer’s protocol. The relative gene expression levels were quantified using the ΔΔCt method. The transcript levels were normalized to those of the housekeeping gene glyceraldehyde-3-phosphate dehydrogenase (GAPDH). Data are presented as the mean ± standard deviation (SD) of three independent experiments.

### 2.5. Gel Electrophoresis of PCR Products

PCR was performed using GoTaq Green Master Mix (Promega, Madison, WI, USA) according to the manufacturer’s protocol. The sequences of the primers used for the evaluation are listed in Table S1. PCR products were loaded on a 2% agarose gel (Agarose S: NIPPON GENE, Tokyo, Japan) with a fluorescent DNA staining reagent (Midori Green Xtra: NIPPON Genetics, Tokyo, Japan) and separated in 1× TAE buffer. Specific bands were detected by the FAS-V gel imaging system (NIPPON Genetics).

### 2.6. Cell Growth Assay

Cell growth was quantified using a RealTime-Glo MT Cell Viability Assay Kit (Promega) according to the manufacturer’s protocol.

### 2.7. Quantification of Apoptosis Induction

To evaluate the activity of caspase-3, APOPCYTO Caspase-3 Fluorometric Assay Kit (MBL, Tokyo, Japan) was used according to the manufacturer’s instructions. Briefly, forty-eight hours after transfection of siRNA, cells were lysed using the lysis buffer supplied with the kit, and the supernatants were collected and incubated with reaction buffer containing dithiothreitol and substrates at 37 °C. To measure caspase-3 activity, the fluorescence counts with a 380 nm excitation filter and a 460 nm emission filter were quantified in a microplate reader. Externalization of phosphatidylserine in the cell membrane, as an indicator of early apoptosis, was quantified using a RealTime-Glo Annexin V Apoptosis Assay Kit (Promega) according to the manufacturer’s protocol.

### 2.8. Antibodies

The anti-KLHDC4 rabbit antibody was purchased from Proteintech (Rosemont, IL, USA). The anti-PARP rabbit antibody was purchased from Cell Signaling Technology (Danvers, MA, USA). The anti-p53 (DO-1) and anti-p21 (F-5) mouse antibodies were purchased from Santa Cruz Biotechnology (Dallas, TX, USA). The pan-actin (ACTN05(C4)) mouse antibodies were purchased from Novus Biologicals (Centennial, CO, USA).

### 2.9. Western Blot Analysis

Whole-cell lysates were obtained at 4 °C with RIPA buffer (150 mM NaCl, 1% NP40, 0.5% sodium deoxycholate, 0.1% sodium dodecyl sulfate (SDS), and 50 mM Tris HCl (pH 8.0)). The samples underwent fractionation by SDS–polyacrylamide gel electrophoresis (SDS–PAGE) and were transferred to Immobilon-P membranes (Merck). Immunoreactive proteins were detected using an enhanced chemiluminescence reagent (ECL Western Blotting Substrate, Thermo Fisher Scientific). Membrane images are presented in Figure S7.

### 2.10. Subcellular Fractionation

Subcellular isolation of RNAs in HCT116 and DLD1 cells was conducted with an RNA Subcellular Isolation Kit (Active Motif, Carlsbad, CA, USA) according to the manufacturer’s instructions. In the cytoplasmic and nuclear fractions, mRNA transcript abundances were quantified by RT–qPCR.

### 2.11. RNA Sequencing (RNA-seq) Analysis

RNA-seq was performed using a NovaSeq 6000 instrument (Illumina, San Diego, CA, USA) according to the manufacturer’s protocol, and data were analyzed as previously described [24]. Briefly, the acquired sequence reads were aligned to the human genome sequence (hg38) using STAR v2.7.10b. The generated Sam files were sorted and converted to bam files with SAMtools v1.16.1. The expression of each gene was quantified and normalized using RSEM v1.3.1. The expression levels of p53 target genes with sufficient expression were converted to Z scores and were subjected to hierarchical clustering analysis based on the average Euclidean distance using gplots v3.1.3 (R package).

### 2.12. Colony Formation Assay

One day after HCT116 and DLD1 cells were transfected with plasmids, the same number of cells was reseeded in three 6 cm dishes. The next day, the medium was replaced with a medium containing puromycin, and then the cells were cultured for 2 weeks. After methanol fixation and air-drying, Giemsa staining (Merck) was performed, and the area of the colonies was quantified using ImageJ.

## 3. Results

### 3.1. High Expression of lncRNA RP11-278A23.1 Is Correlated with Poor Prognosis in Colorectal Cancers

**Figure 1:**
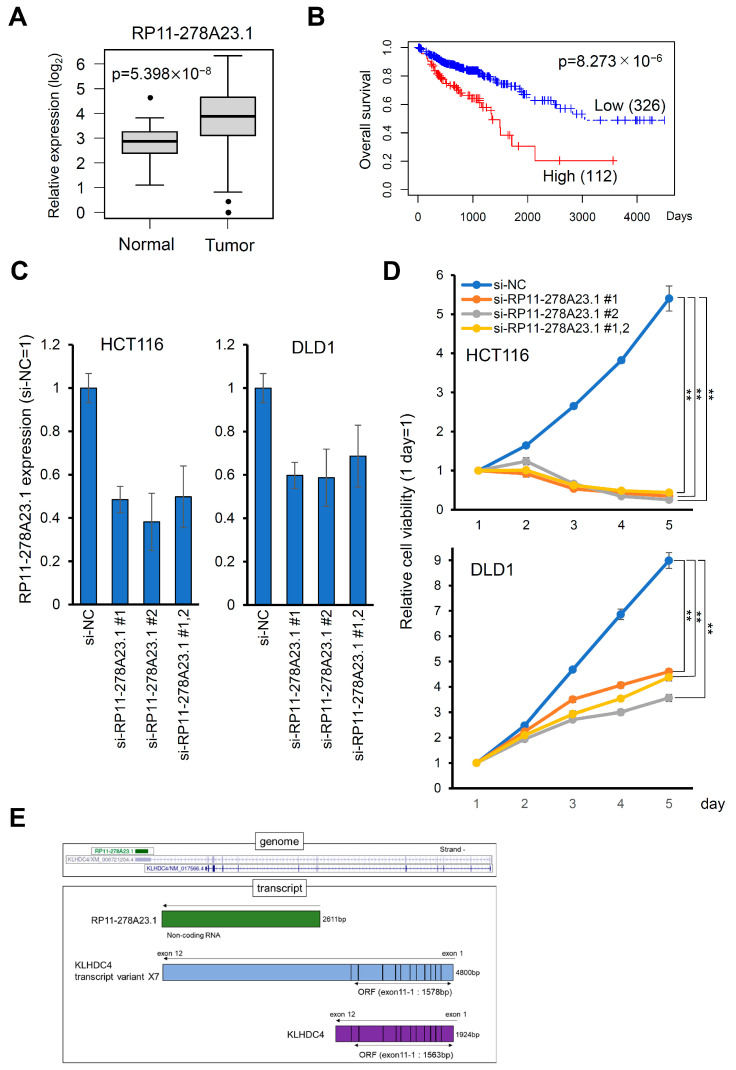
RP11-278A23.1 expression correlates with poor prognosis and cell proliferation in colorectal cancers. (**A**) Comparison of RP11-278A23.1 expression between colorectal cancer tissues and normal tissues. RNA-seq data of colorectal cancer tissues and normal tissues in the TCGA database were analyzed, and RP11-278A23.1 expression levels are presented in boxplots. (**B**) Correlation between RP11-278A23.1 expression and prognosis in colorectal cancer patients. The correlation between RP11-278A23.1 and survival in the TCGA dataset was analyzed, and survival curves were generated using the Kaplan–Meier method. The survival rates for patients with high and low RP11-278A23.1 expression are plotted as red and blue lines, respectively. The number of patients in each group is shown in parentheses. *p* values were calculated using the log-rank test. HCT116 and DLD1 cells were transfected with siRNA-RP11-278A23.1 #1, siRNA-RP11-278A23.1 #2, or a negative control siRNA (si-NC). Forty-eight hours after transfection, mRNA was extracted, and RP11-278A23.1 expression was analyzed by RT–qPCR (**C**). Under the same conditions, cell growth was quantified (**D**). The error bars indicate the SDs. The double asterisk indicates a *p*-value of <0.01, as determined by Welch’s two-sided *t*-test. (**E**) Schematic diagram of RP11-278A23.1, KLHDC4, and KLHDC4 transcript variant X7.

To identify lncRNAs contributing to colorectal cancers, we investigated lncRNAs that were highly expressed in colorectal cancer tissues compared to normal tissues and were associated with poor prognosis in the corresponding high-expression groups using a dataset from TCGA. We selected lncRNAs whose expression was increased more than two-fold in colorectal cancers compared to normal tissues and whose *p*-value was less than 1 × 10^−5^ in the overall survival analysis. Among them, we focused on lncRNA RP11-278A23.1, which was not reported before. RP11-278A23.1 expression was significantly increased in colorectal cancer tissues compared with normal tissues (Figure 1A). Furthermore, in colorectal cancer, patients in the high RP11-278A23.1 expression group were found to have significantly poorer prognoses in terms of not only overall survival but also disease-free survival, disease-specific survival, and progression-free survival (Figure 1B and Figure S1A). Next, in the survival analysis of the other 32 cancer types, the prognosis was found to be significantly poorer in the high RP11-278A23.1 expression group in adrenocortical cancer, esophageal cancer, clear cell renal cell carcinoma, malignant mesothelioma, cholangiocarcinoma, and prostate cancer (Figure S1B).

### 3.2. Knockdown of RP11-278A23.1 Suppresses Cell Growth and Induces Apoptosis

**Figure 2:**
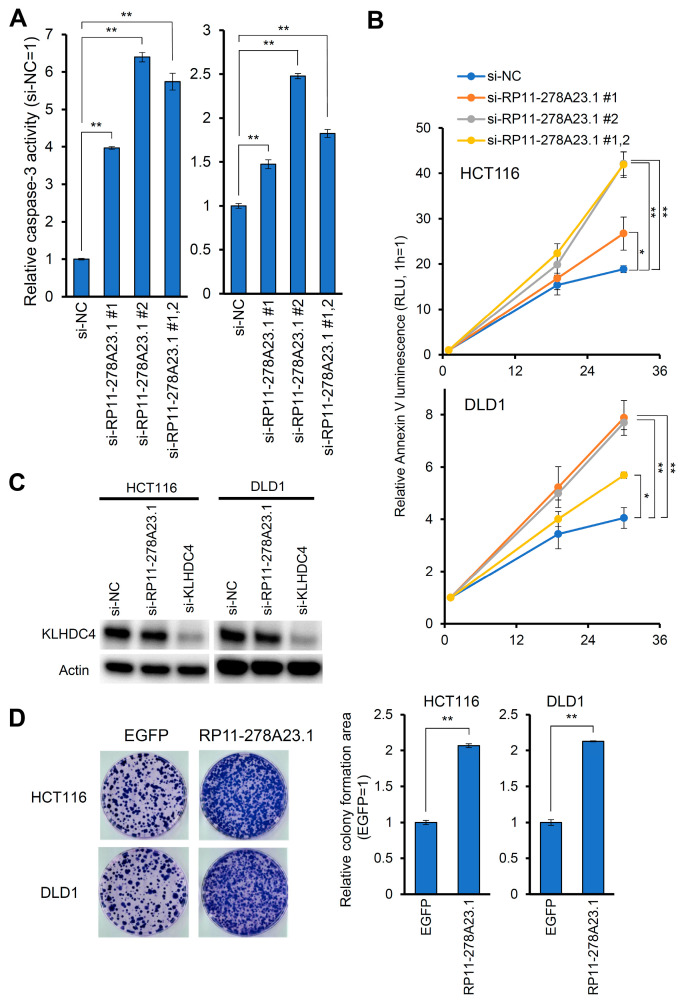
Knockdown of RP11-278A23.1 induces apoptosis. (**A**) HCT116 and DLD1 cells were transfected with siRNA-RP11-278A23.1 #1, siRNA-RP11-278A23.1 #2, or a negative control siRNA (si-NC). Forty-eight hours after transfection, Caspase-3 activity was quantified. (**B**) Under the same transfection conditions as in (**A**), the proportion of annexin-V-positive cells was quantified at the indicated times (hours). (**C**) HCT116 and DLD1 cells were transfected with siRNA-RP11-278A23.1 (#1, #2), siRNA-KLHDC4, or a negative control siRNA (si-NC). Forty-eight hours after transfection, the protein expression of KLHDC4 and actin as a control was confirmed by Western blotting. Membrane images are presented in Figure S7. (**D**) HCT116 and DLD1 cells were transfected with plasmids expressing RP11-278A23.1 or EGFP and selected with puromycin for two weeks. The assay was performed in triplicate. Representative staining is presented (**left**), and the colony formation area was quantified (**right**). The error bars indicate the SDs. The single and double asterisks indicate *p*-values of <0.05 and 0.01, respectively, as determined by Welch’s two-sided *t*-test.

To investigate the biological function of RP11-278A23.1, we knocked down RP11-278A23.1 with siRNAs in the colon cancer cell lines HCT116 and DLD1 (Figure 1C) and examined the effect on cell growth. RP11-278A23.1 knockdown significantly suppressed cell growth (Figure 1D). Next, to investigate whether RP11-278A23.1 is involved in apoptosis, we knocked down RP11-278A23.1 and performed a Caspase-3 assay and Annexin V analysis to evaluate these indicators of apoptosis. The knockdown of RP11-278A23.1 increased Caspase-3 activity (Figure 2A) and Annexin V binding (Figure 2B).

### 3.3. RP11-278A23.1 Function Is Independent of the KLHDC4 Protein

In the National Center for Biotechnology Information (NCBI) database, we found a continuous transcript between RP11-278A23.1 and the adjacent coding gene KLHDC4, named KLHDC4 transcript variant X7 (KLHDC4-X7) (Figure 1E). We confirmed that KLHDC4-X7 is also expressed in colorectal cancer cell lines by RT–PCR using KLHDC4-X7-specific primers (Figures S2 and S3A) and Sanger sequencing. It was reported that the high expression of KLHDC4 is a poor prognostic factor in nasopharyngeal carcinoma [25]. Therefore, we confirmed whether si-RP11-278A23.1 affects KLHDC4 protein expression by Western blotting. The transfection of either si-RP11-278A23.1 or si-KLHDC4 significantly reduced KLHDC4-X7 RNA expression (Figure S3A,B), but si-RP11-278A23.1 did not reduce KLHDC4 protein expression (Figure 2C). Furthermore, the overexpression of KLHDC4-X7 increased KLHDC4 mRNA expression (Figure S4A) but not KLHDC4 protein expression (Figure S4B), even though the sequence of KLHDC4-X7 in the overexpression vector contained the KLHDC4 ORF. The knockdown of KLHDC4-X7 by si-KLHDC4, followed by the restoration of KLHDC4 protein expression with the KLHDC4-expressing plasmid, resulted in increased apoptotic activity (Figure S4C). In addition, the overexpression of RP11-278A23.1 significantly increased colony formation compared to that of control cells (Figure 2D). These results suggest that RP11-278A23.1 and KLHDC4-X7, which have sequences identical to that of RP11-278A23.1, function as noncoding RNAs and promote the growth and inhibit the apoptosis of colorectal cancer cells independently of the KLHDC4 protein.

### 3.4. RP11-278A23.1 and KLHDC4-X7 Localize to the Nucleus

**Figure 3:**
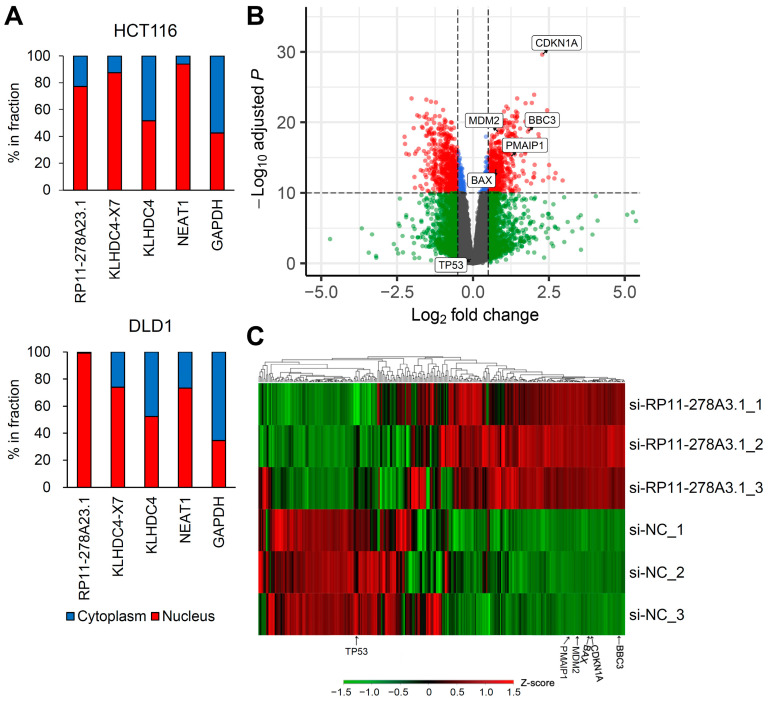
Effect of RP11-278A23.1 knockdown on gene expression. (**A**) In HCT116 and DLD1 cells, the proportions of RP11-278A23.1, KLHDC4-X7, and KLHDC4 mRNA in the nucleus and cytoplasm were quantified by qPCR. NEAT1 and GAPDH were used as controls for nuclear and cytoplasmic mRNA, respectively. HCT116 cells were transfected with si-RP11-278A23.1 (#1, #2) or a negative control siRNA (si-NC). Forty-eight hours after transfection, mRNA was extracted and used for RNA-seq analysis. (**B**) Fold changes (si-RP11-278A23.1/si-NC) and adjusted *p* values are presented in a volcano plot. The vertical and horizontal dashed lines indicate 0.5 in log_2_ fold change and *p* = 1 × 10^−10^, respectively. (**C**) A total of 293 genes selected as p53 targets (Table S4) were analyzed using hierarchical clustering. In the heatmap, red and green indicate positive and negative Z scores, respectively.

Many lncRNAs are reported to be localized in the nucleus due to their functions [26]. To examine the subcellular localization of RP11-278A23.1 and KLHDC4-X7, we quantified their abundances in the nuclear and cytoplasmic fractions by qPCR. Both were abundantly in the nuclear fraction, consistent with the general localization of lncRNAs (Figure 3A).

### 3.5. RP11-278A23.1 Knockdown in p53-Wild-Type Colorectal Cancer Cells Alters p53 Target Gene Expression

**Figure 4:**
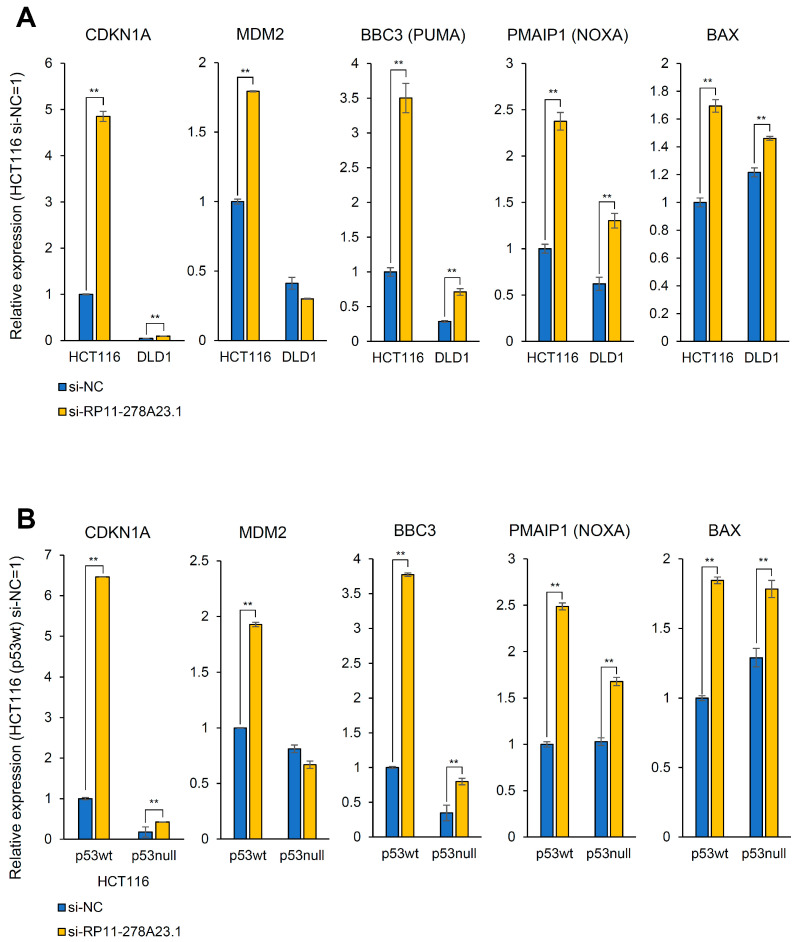
Changes in the expression of p53 targets and apoptosis-related genes after RP11-278A23.1 knockdown. (**A**) HCT116 and DLD1 cells were transfected with si-RP11-278A23.1 (#1, #2) or a negative control siRNA (si-NC). Forty-eight hours after transfection, mRNA was extracted and used for RNA-seq analysis. The relative mRNA expression of the indicated genes is presented. (**B**) HCT116 (p53+/+) and HCT116 (p53−/−) cells were transfected under the same transfection conditions described in (**A**). Forty-eight hours after transfection, mRNA was extracted and used for RNA-seq analysis. The relative mRNA expression of the indicated genes is presented. The averages of three experiments are indicated as relative expression levels, with si-NC = 1. The error bars indicate the SDs. NC, negative control. The double asterisk indicates a *p*-value of <0.01, as determined by Welch’s two-sided *t*-test.

To comprehensively examine the effect of RP11-278A23.1 on gene transcription, RNA-seq was performed in RP11-278A23.1-knockdown HCT116 cells. The expression of p21, a p53 target gene, was significantly upregulated in RP11-278A23.1-knockdown cells compared with control cells (Figure 3B). To confirm the broad effect of gene expression change, we performed gene ontology analysis by DAVID (https://david.ncifcrf.gov/ (accessed on 10 February 2024)) [27] using the top 100 differentially expressed genes between RP11-278A23.1-knockdown and control HCT116 cells (Table S2). The analysis indicated the p53 signaling pathway as a top cluster (Table S3). Since HCT116 cells have wild-type p53 and p21 is the target gene of p53, we performed Western blotting to confirm whether p53 is activated. RP11-278A23.1 knockdown increased the expression of both the p53 and p21 proteins (Figure S5A). Therefore, we selected 293 p53 target genes with sufficient expression (Table S4) for hierarchical clustering analysis [28] and found that RP11-278A23.1 knockdown resulted in the generation of clusters of genes with up- and downregulated expression, which included many apoptosis-related genes (Figure 3C). Clusters of genes with upregulated expression relative to that in control cells contained MDM2 and several proapoptotic genes, such as BBC3 (PUMA) and PMAIP1 (NOXA) (Figure 4A, HCT116). However, p53 expression was not affected at the transcriptional level (Figure S5B). Furthermore, RP11-278A23.1 expression was inversely correlated with CDKN1A and MDM2 expression in the TCGA-COAD dataset (Figure S6). These results suggest that RP11-278A23.1 knockdown affects p53 target selectivity in p53 wild-type HCT116 cells, which is one reason for the suppression of cell growth and the induction of apoptosis.

### 3.6. RP11-278A23.1 Also Affects p53-Independent Pathways

**Figure 5:**
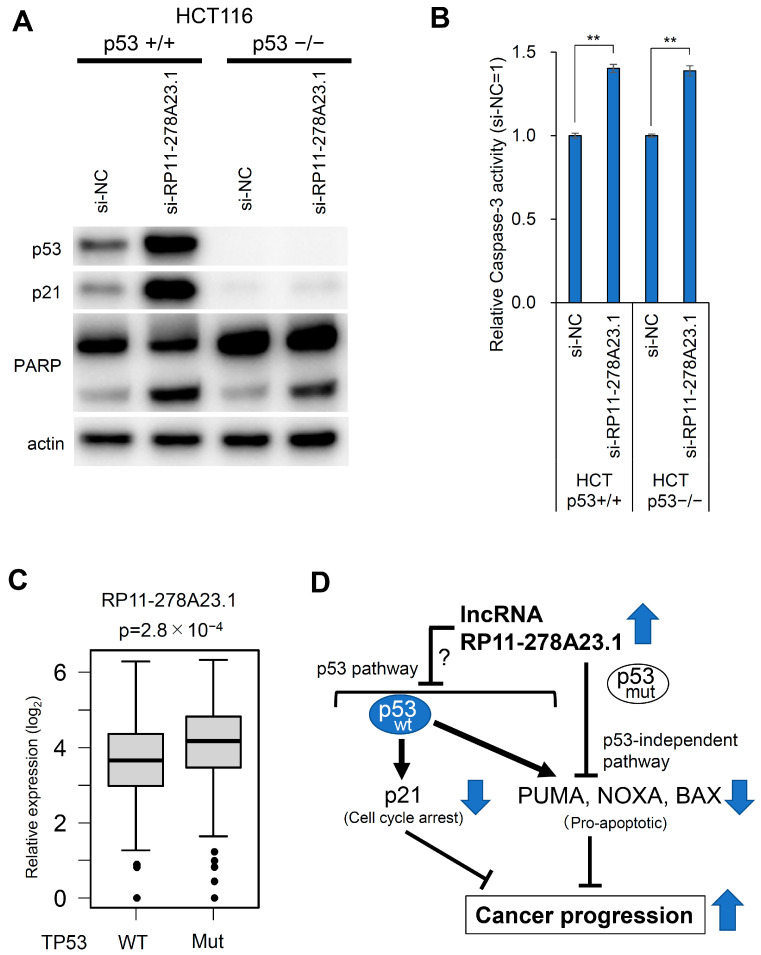
The effect of RP11-278A23.1 knockdown on apoptosis in p53-knockout cells. Forty-eight hours after transfection of si-NC and si-RP11-278A23.1 into HCT116 (p53+/+) and HCT116 (p53−/−) cells, whole-cell lysates were obtained, and Western blotting (**A**) and a Caspase-3 assay (**B**) were performed. Membrane images are presented in Figure S7. The error bars indicate the SDs. The double asterisk indicates a *p*-value of <0.01, as determined by Welch’s two-sided *t*-test. NC, negative control. (**C**) RNA-seq data of colorectal cancers in a TCGA dataset (TCGA-COAD) were divided into p53 wild-type (WT, 235 cases) and p53 mutant (Mut, 223 cases) groups and were then analyzed. RP11-278A23.1 expression levels are presented in boxplots. (**D**) Schematic representation of the roles of RP11-278A23.1 in colorectal cancers. The blue arrows pointing up and down indicate increases and decreases, respectively, in expression/activity.

In p53 wild-type HCT116 cells, the knockdown of RP11-278A23.1 affected p53 target selectivity, resulting in the suppression of cell growth and the induction of apoptosis. However, even though DLD1 cells harbor a mutation in p53, the knockdown of RP11-278A23.1 was followed by cell growth inhibition and apoptosis induction as robust as those in HCT116 cells, suggesting that a p53-independent pathway may also be involved. Therefore, we knocked down RP11-278A23.1 in DLD1 cells and performed RNA-seq. Similar to the observations in HCT116 cells, the expression of the proapoptotic genes BBC3, PMAIP1, and BAX was upregulated in DLD1 cells (Figure 4A). In addition, we knocked down RP11-278A23.1 in p53-wild-type HCT116 cells (HCT116 (p53+/+)) and p53-knockout HCT116 cells (HCT116 (p53−/−)) and performed RNA-seq. RP11-278A23.1 knockdown also upregulated BBC3, PMAIP1, and BAX expression in HCT116 (p53−/−) cells (Figure 4B), although the changes were weaker than those in HCT116 (p53+/+) cells. In these cells, p53 expression was not affected at the transcriptional level (Figure S5B). Therefore, the knockdown of RP11-278A23.1 in p53-mutant cells also induces the expression of these apoptosis-related genes via a p53-independent pathway. Furthermore, the knockdown of RP11-278A23.1 was followed by the cleavage of PARP, an indicator of apoptosis, as shown by Western blotting (Figure 5A) and by increased Caspase-3 activity (Figure 5B) in not only HCT116 (p53+/+) but also HCT116 (p53−/−) cells, consistent with the results in DLD1 cells, which harbor mutated p53 (Figure 2A). Interestingly, RP11-278A23.1 expression was significantly higher in colorectal cancers with mutant p53 than in those with wild-type p53 (Figure 5C). These results suggest that RP11-278A23.1 contributes to colorectal cancer progression through a p53-independent pathway.

## 4. Discussion

In this study, the screening of lncRNAs through expression and survival analyses in a TCGA dataset suggested that RP11-278A23.1 may be involved in the development of colorectal cancer. The survival rate in the high RP11-278A23.1 expression group was decreased in colorectal cancer (Figure 1B and Figure S1A), as well as adrenocortical cancer, esophageal cancer, clear cell renal cell carcinoma, malignant mesothelioma, cholangiocarcinoma, and prostate cancer (Figure S1B). Therefore, the high expression of RP11-278A23.1 may be a poor prognostic factor in cancers. Furthermore, the knockdown of RP11-278A23.1 increased apoptotic activity, as shown by findings such as the increases in Caspase-3 activity and Annexin V binding (Figure 2A,B), in addition to suppressing cell growth in colorectal cancer cell lines (Figure 1D).

However, we identified a continuous transcript between RP11-278A23.1 and the adjacent coding gene KLHDC4, which was annotated as KLHDC4-X7. Then, we confirmed that KLHDC4-X7 is expressed in colorectal cancer cell lines (Figure S3A). However, RT-qPCR and Western blot analyses revealed that the translation of KLHDC4-X7 to the KLHDC4 protein was minimal (Figure 2C and Figure S4B). Moreover, subcellular localization analysis of RP11-278A23.1 and KLHDC4-X7 showed that both were abundant in the nuclear fraction (Figure 3A). Since most lncRNAs function in the nucleus, they are reported to be abundant in the nuclear fraction [26]. Thus, this result is consistent with the general localization of lncRNAs. The knockdown of KLHDC4-X7 by si-KLHDC4 followed by restoration of KLHDC4 protein expression with the KLHDC4-expressing plasmid resulted in increased apoptotic activity (Figure S4C). Therefore, it is considered that KLHDC4-X7, which includes the identical sequence as RP11-278A23.1, also works as a nuclear lncRNA rather than an mRNA, even though KLHDC4-X7 has an open reading frame. These results suggest that RP11-278A23.1 contributes to colorectal cancer progression.

To comprehensively examine differentially expressed genes due to the knockdown of RP11-278A23.1, RNA-seq was performed in HCT116 cells. In the volcano plot, the expression of p21, a p53 target gene, was shown to be significantly upregulated (Figure 3B). Western blotting also showed increases in p53 and p21 protein expression (Figure S5A). p21 is a cyclin-dependent kinase inhibitor that functions to block cell cycle progression during the G1 phase [29]. Thus, one possible reason that cell growth is inhibited by the knockdown of RP11-278A23.1 could be cell cycle arrest induced by transcriptional activation of p21. Since HCT116 cells harbor wild-type p53 and p21, a target gene of p53, we selected 293 p53 target genes [28] with sufficient expression for hierarchical clustering analysis (Figure 3C). The results showed that RP11-278A23.1 knockdown resulted in the generation of clusters of genes with up- and down-regulated expression, which included many apoptosis-related genes. These results suggest that the activation of apoptosis by RP11-278A23.1 knockdown is due not only to p53 activation but also to transcriptional changes in apoptosis-related genes downstream of p53 resulting from an effect on p53 target selectivity. Therefore, the effect of RP11-278A23.1 knockdown on p53 target selectivity in p53-wild-type HCT116 cells may be one reason for the inhibition of cell growth and the induction of apoptosis (Figure 5D, left; wild-type 53 pathway).

However, although DLD1 cells harbor a mutation in p53, the knockdown of RP11-278A23.1 was followed by inhibition of cell growth and induction of apoptosis as robust as those observed in HCT116 cells. Therefore, we knocked down RP11-278A23.1 in DLD1 cells and performed RNA-seq. Similar to the observations in HCT116 cells, the expressions of the proapoptotic genes BBC3 (PUMA), PMAIP1 (NOXA), and BAX were upregulated in DLD1 cells, although the expression of p21 was barely upregulated (Figure 4A). 

In addition, we knocked down RP11-278A23.1 in p53-wild-type HCT116 cells (HCT116 (p53+/+)) and p53-knockout HCT116 cells (HCT116 (p53−/−)) and performed RNA sequencing and RT-qPCR. The results showed that RP11-278A23.1 knockdown also upregulated BBC3, PMAIP1, and BAX expression in HCT116 (p53−/−) cells (Figure 4B). Therefore, the knockdown of RP11-278A23.1 in p53-mutant cells also induces the expression of these apoptosis-related genes via a p53-independent pathway. Furthermore, the knockdown of RP11-278A23.1 in both HCT116 (p53+/+) and HCT116 (p53−/−) cells was followed by cleavage of PARP, an indicator of apoptosis, as shown by Western blotting (Figure 5A) and by increased Caspase-3 activity, as shown by the Caspase-3 assay (Figure 5B). These results suggest that RP11-278A23.1 knockdown induces apoptosis also through a p53-independent pathway (Figure 5D, right; mutated p53 pathway). 

Several lncRNAs were reported to contribute to colorectal cancer through various mechanisms. For example, HOTAIR interacts with the polycomb repressive complex 2 (PRC2), leading to changes in cell epigenetics and gene expression [11]. Meanwhile, MALAT-1 modulates pre-mRNA splicing and directly regulates gene expression by binding it to chromatin [30]. Additionally, CCAT1 and CCAT2 exhibit oncogenic functions through a process known as miRNA sponging [31,32]. However, there are few reports of altering the expression of p53 target genes, as RP11-278A23.1 exhibits. Considering this, it seems that RP11-278A23.1 could be a potentially promising and effective therapeutic target.

Targeting lncRNAs therapeutically is still challenging. Among various strategies, antisense oligonucleotide (ASO) is one of the promising candidates for targeting oncogenic lncRNAs. ASOs are single-stranded oligonucleotides that have target-specific complement sequences and induce RNase H-mediated degradation of target lncRNAs. ASO may be more effective than siRNAs for lncRNA targets because the secondary structure of lncRNA might block siRNA association. Locked nucleic acid (LNA) nucleotides also bind to target lncRNAs, leading to their degradation via RNAse H-dependent mechanism. Gapmers, oligonucleotides composed of short DNA strands flanked by RNA mimic LNAs, are also promising candidates for targeting oncogenic lncRNAs. These strategies may be useful for the therapeutic targeting of RP11-278A23.1.

## 5. Conclusions

The long non-coding RNA RP11-278A23.1 contributes to colorectal cancer progression by promoting cell growth and inhibiting apoptosis, and this finding suggests that this lncRNA may be a useful therapeutic target.

## Data Availability

The datasets generated and/or analyzed during the current study are available from the corresponding author upon reasonable request.

## Supplementary Materials

The following supporting information can be downloaded at: https://www.mdpi.com/article/10.3390/cancers16050882/s1, Figure S1: RP11-278A23.1 expression correlates with poor prognosis in cancers; Figure S2: Schematic diagram of RP11-278A23.1, KLHDC4, and KLHDC4 transcript variant X7; Figure S3: Knockdown of RP11-278A23.1, KLHDC4, and KLHDC4-X7 by siRNA; Figure S4: Confirmation of KLHDC4 protein expression; Figure S5: The effect of RP11-278A23.1 knockdown on p53 and p21 expression; Figure S6: The correlation between RP11-278A23.1 and CDKN1A/MDM2 expression; Figure S7: Membrane images of Western blotting; Figure S8: Membrane images of Western blotting in supplementary figures; Table S1: Primer sequences for PCR; Table S2: The top 100 differentially expressed genes between RP11-278A23.1-knockdown and control HCT116 cells.; Table S3: Gene ontology analysis of the top 100 differentially expressed genes by RP11-278A23.1 knockdown.; Table S4: The gene list for clustering analysis.
