# Supplementary material for: Long Noncoding RNA RP11-278A23.1, a Potential Modulator of p53 Tumor Suppression, Contributes to Colorectal Cancer Progression
Source: Cancers (Basel). 2024 Feb 22;16(5):882. doi: 10.3390/cancers16050882 (PMC10931219; doi:10.3390/cancers16050882)
Supplement: Supplementary file 1 [file cancers-16-00882-s001.zip › supplementary Figures.pdf]

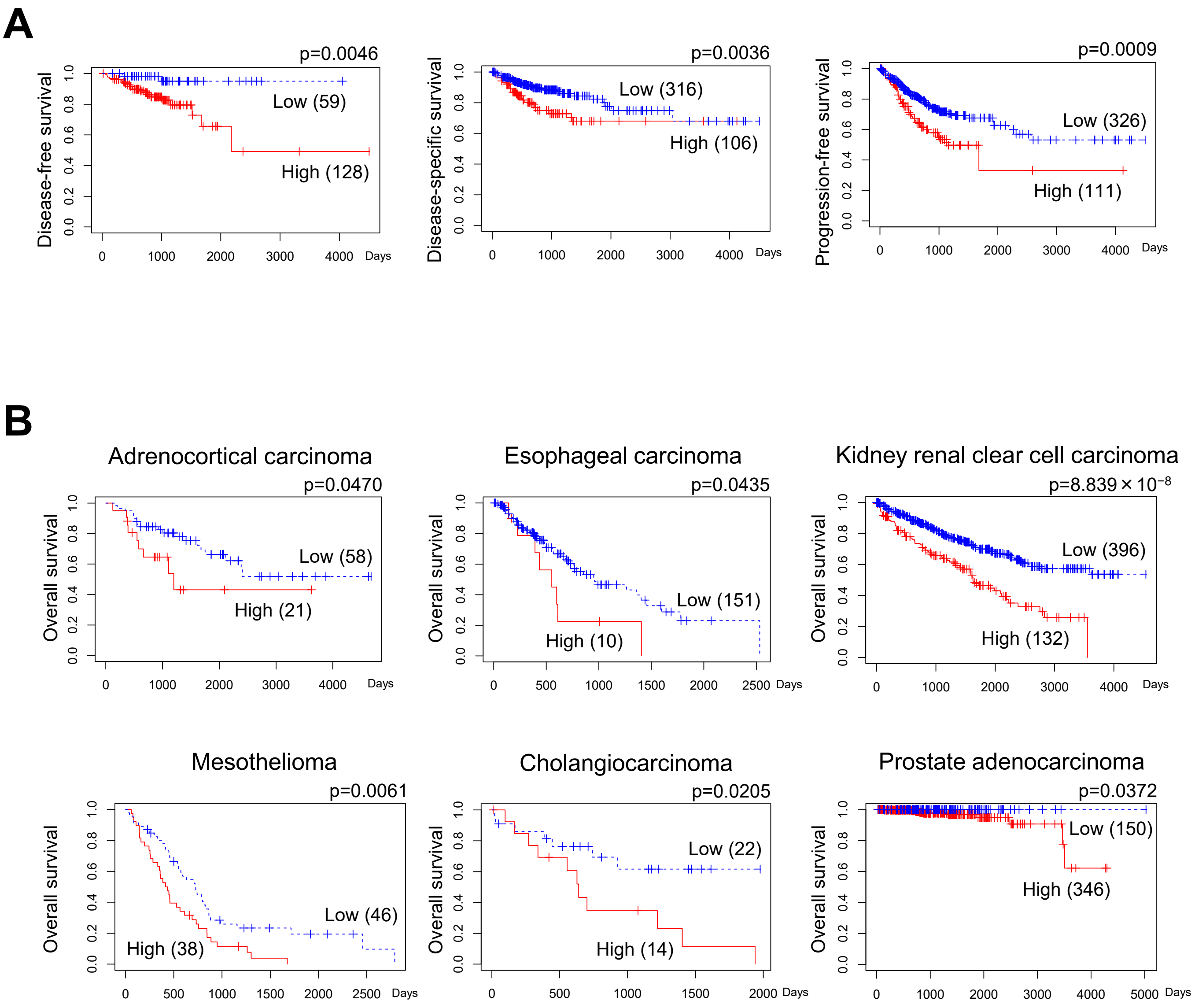

Figure S1

RP11-278A23.1 expression correlates with poor prognosis in cancers.

The indicated survival curves in colorectal cancer (A) and overall survival curves in the indicated cancers (B) were plotted using the Kaplan–Meier method. These curves were generated using data from the TCGA dataset. The survival rates for patients with high and low lncRNA expression are plotted as red and blue lines, respectively. The number of patients in each group is shown in parentheses. P values were calculated by the log-rank test.

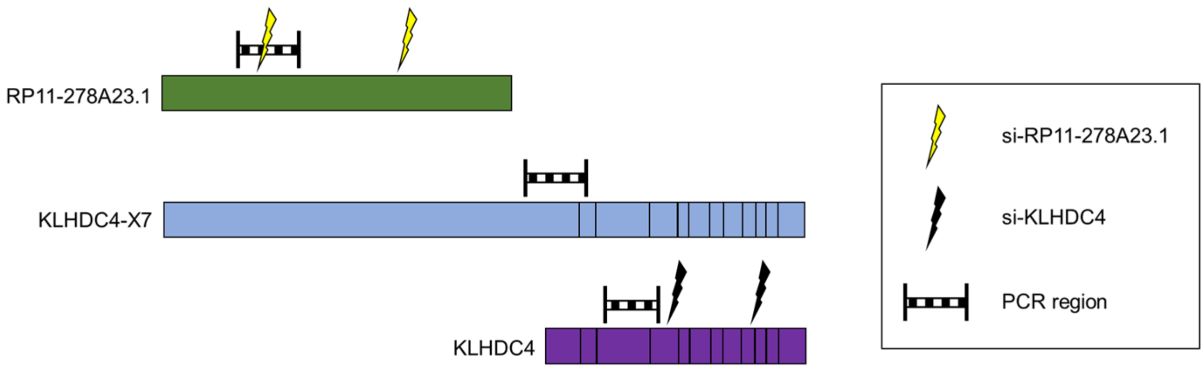

Figure S2

Schematic diagram of RP11-278A23.1, KLHDC4, and KLHDC4 transcript variant X7.

The target sites in si-RP11-278A23.1 and si-KLHDC4 and the PCR target regions for each transcript are shown.

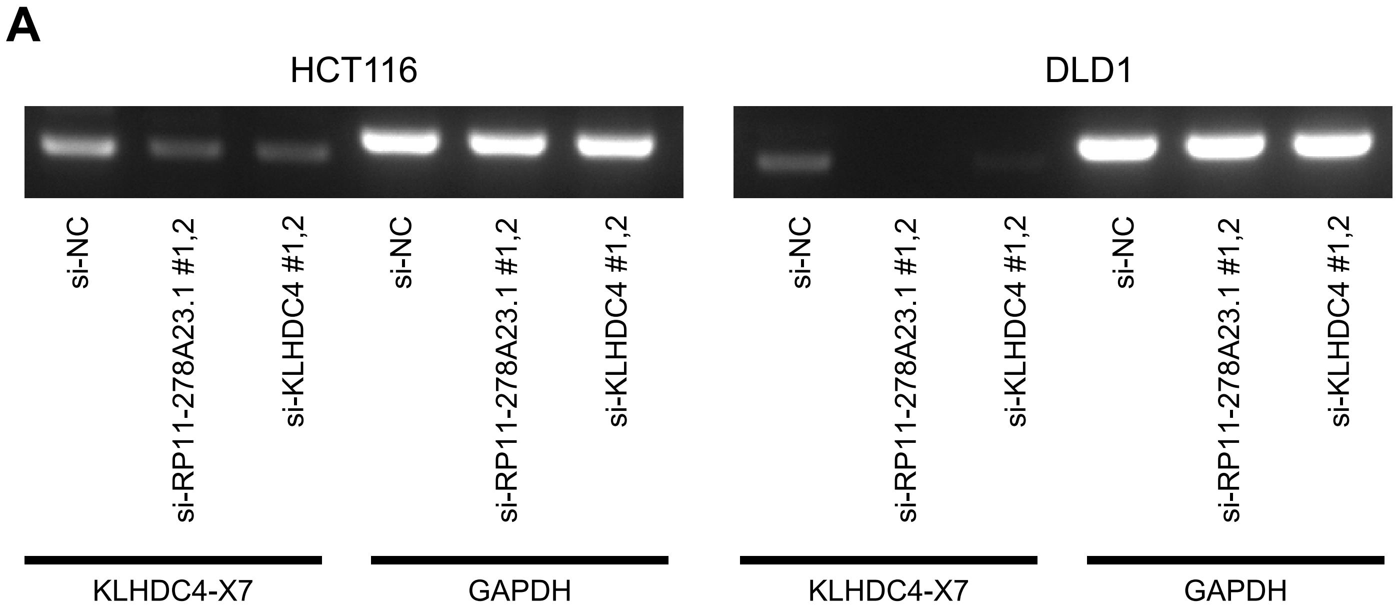

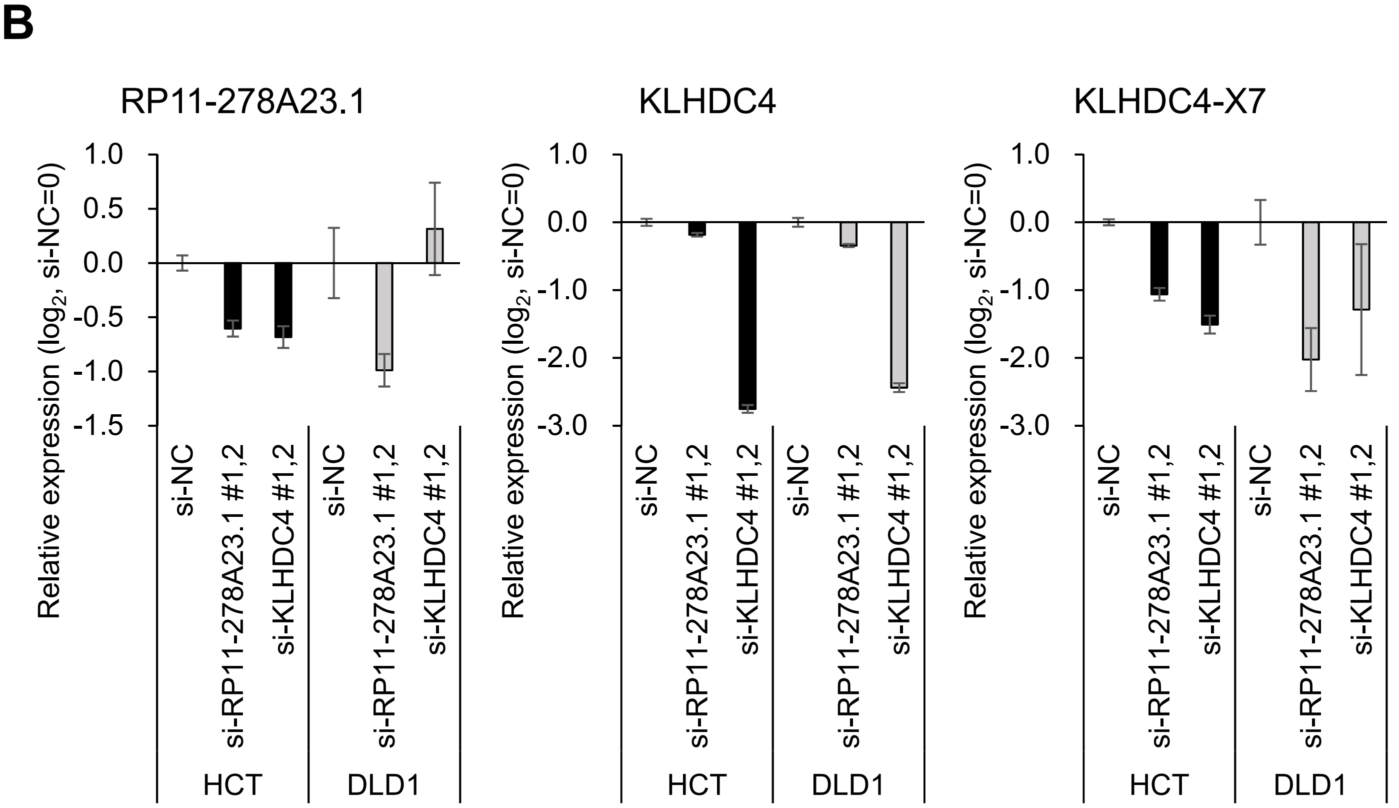

Figure S3

Knockdown of RP11-278A23.1, KLHDC4, and KLHDC4-X7 by siRNA.

Forty-eight hours after transfection of si-NC, si-RP11-278A23.1, and si-KLHDC4, RT–PCR was performed with KLHDC4-X7-specific primers (A). RT–qPCR was also performed (B). The averages of three experiments are indicated as $\log_2$ values with si-NC = 0. The error bars indicate the SDs. NC, negative control.

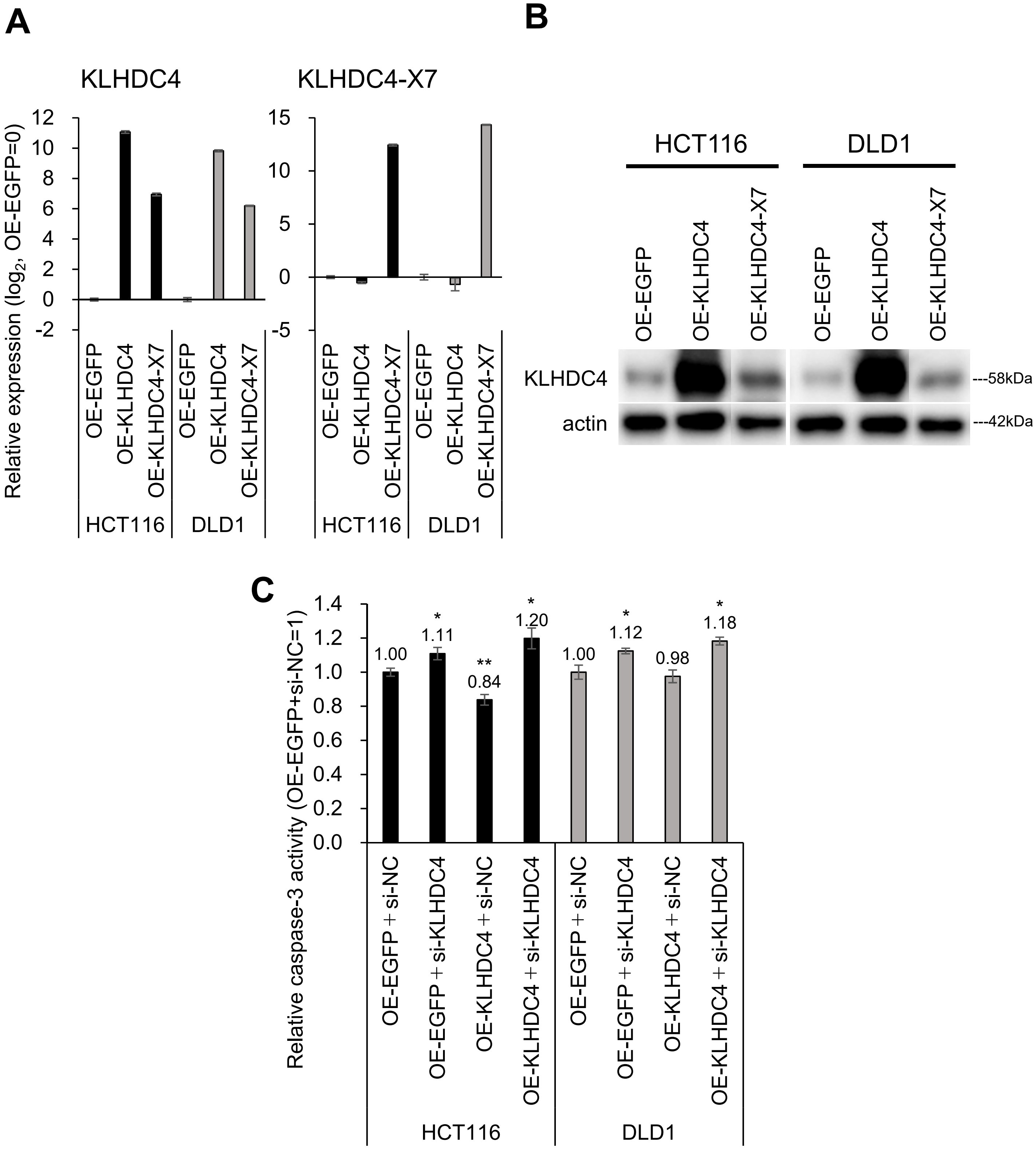

Figure S4

Confirmation of KLHDC4 protein expression.

Twenty-four hours after transfection of the EGFP (control), KLHDC4, and KLHDC4-X7 plasmids, RT–qPCR (A) and Western blotting (B) were performed. For RT–qPCR data, the averages of three experiments are indicated as $\log_2$ values with OE-EGFP = 0. The error bars indicate the SDs.

(C) Forty-eight hours after cotransfection of the siRNA and plasmid, Caspase-3 activity was quantified. The error bars indicate the SDs. The single and double asterisks indicate significance levels of $p < 0.05$ and $p < 0.01$, respectively (Welch's two-sided *t*-test).

NC, negative control. OE, overexpression

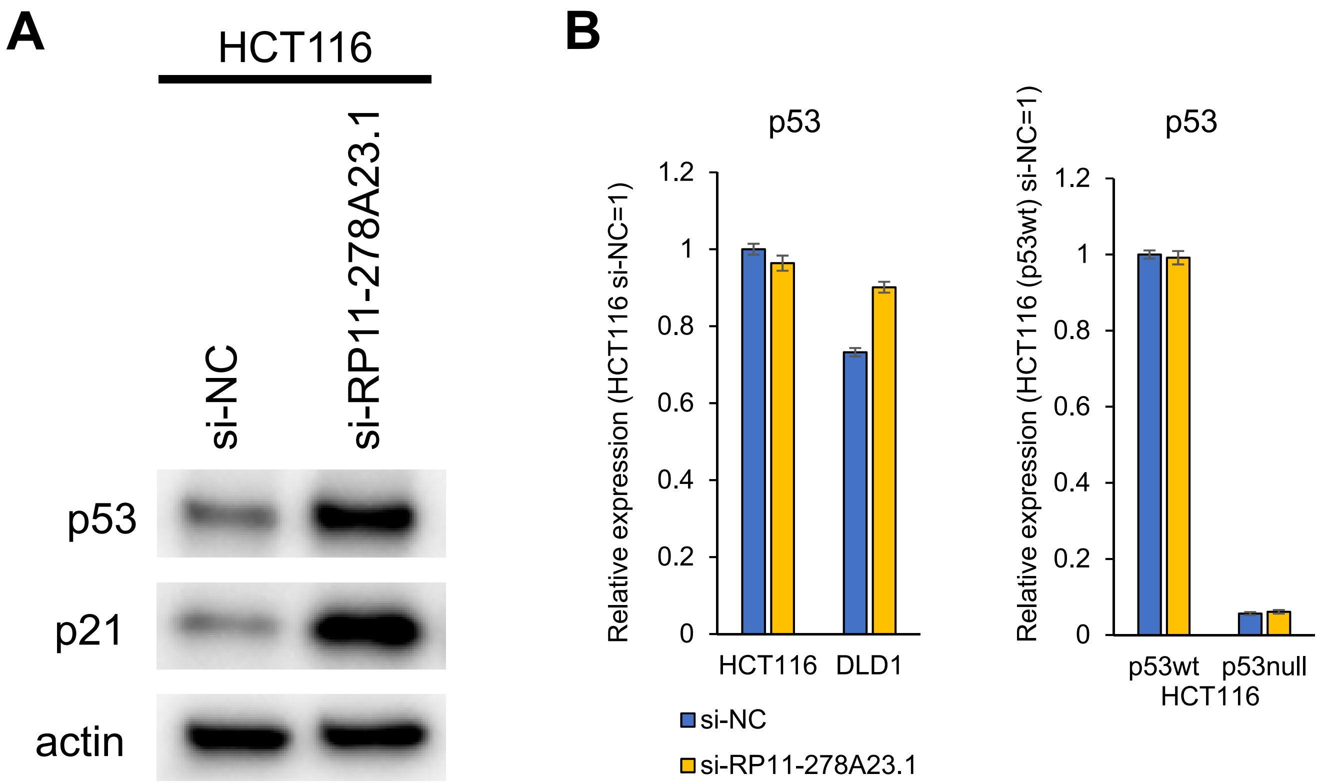

Figure S5

The effect of RP11-278A23.1 knockdown on p53 and p21 expression.

(A) Forty-eight hours after transfection of si-NC or si-RP11-278A23.1 into HCT116 cells, whole-cell lysates were obtained, and Western blotting was performed.

(B) Relative mRNA expression of p53 based on the RNA-seq data in HCT116, DLD1, HCT116 (p53+/+) (p53wt) and HCT116 (p53−/−) (p53null) cells. The error bars indicate the SDs.

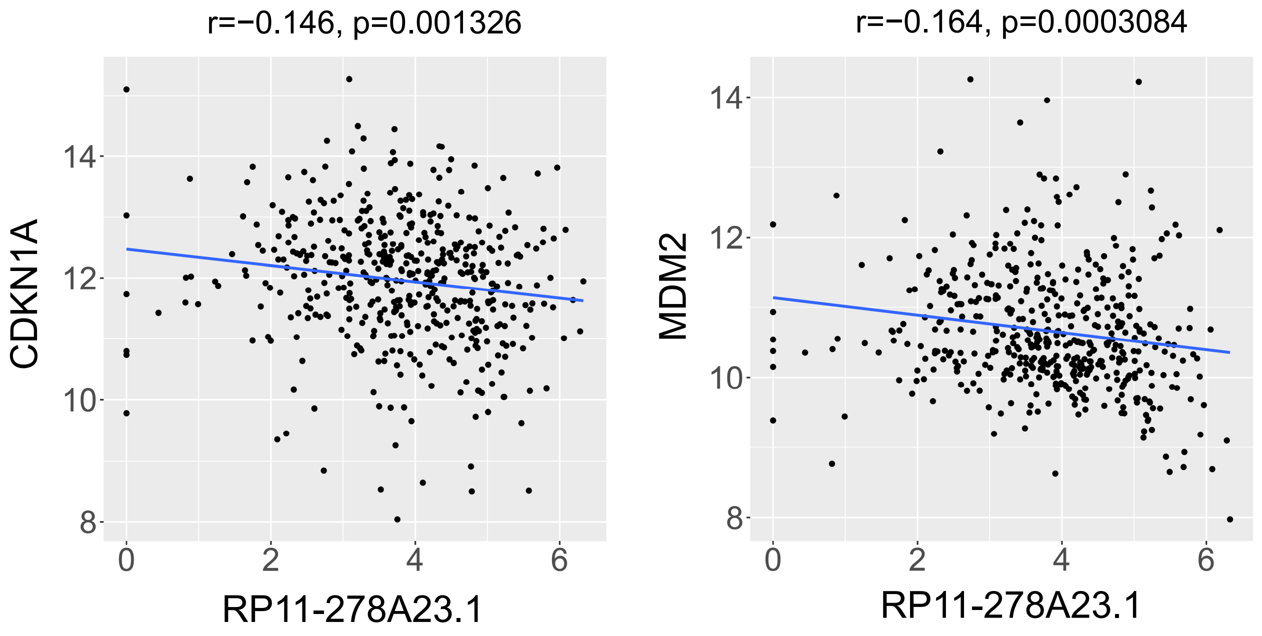

Figure S6

The correlation between RP11-278A23.1 and CDKN1A/MDM2 expression.

The expression of the two indicated genes in TCGA-COAD dataset is presented as a scatter plot. Correlation coefficient (r) and p-value were calculated.

Fig. 2C

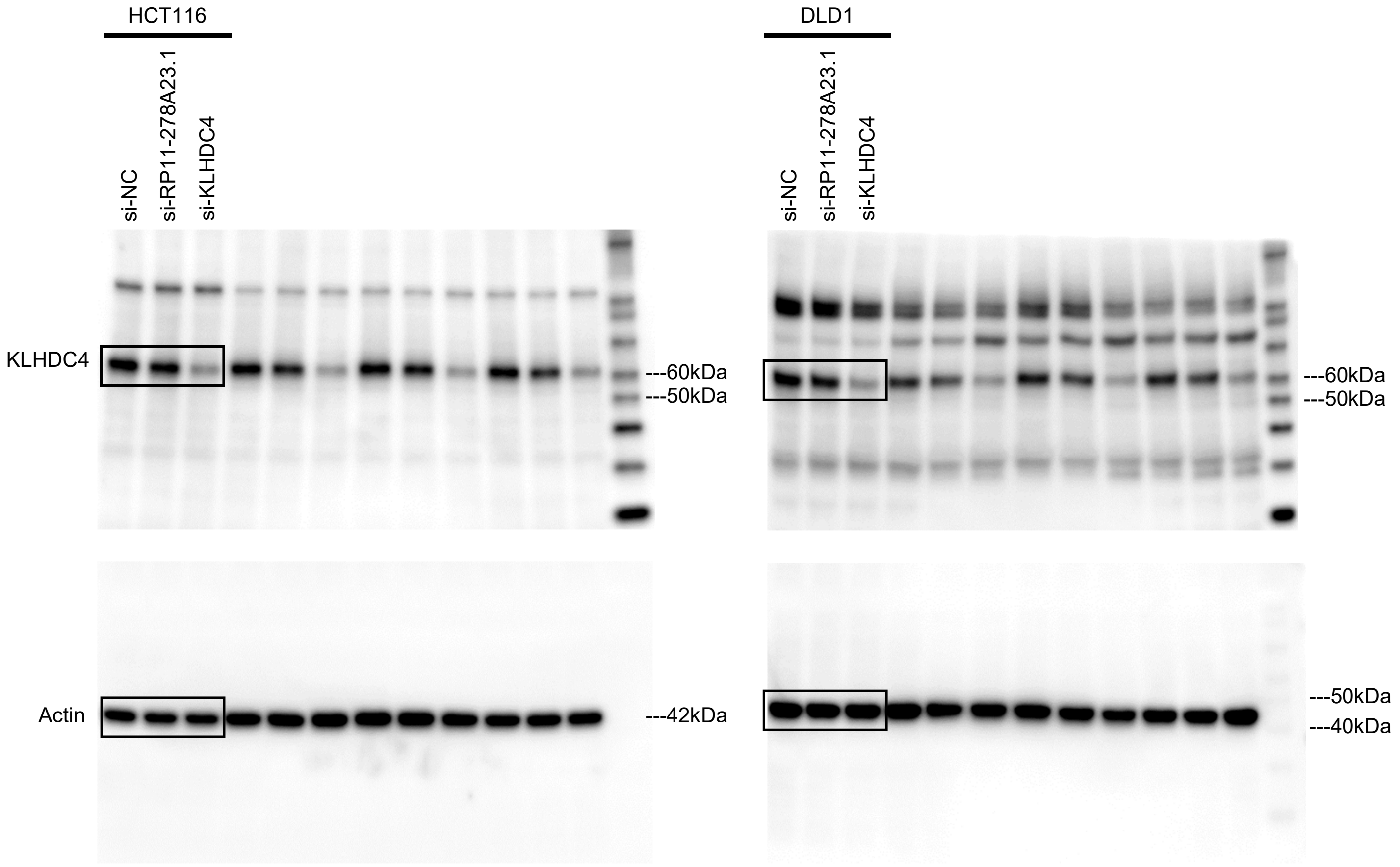

Fig. 5A

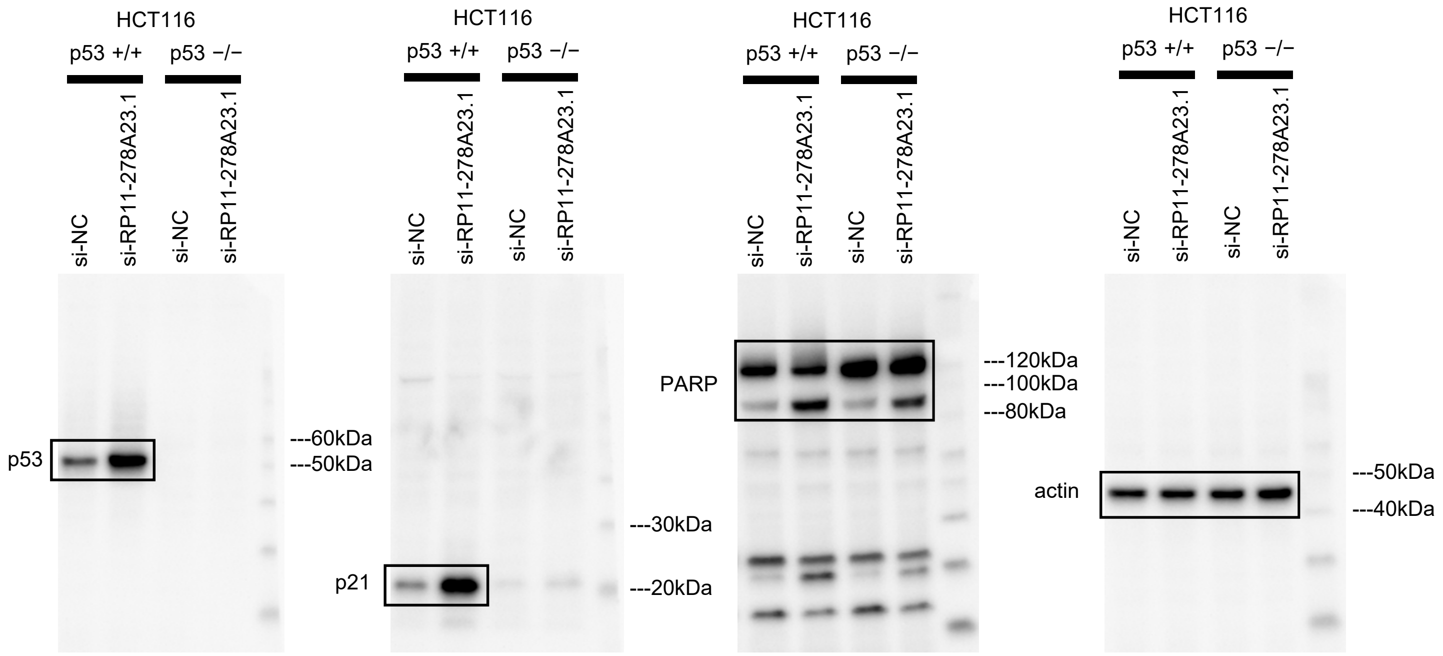

Figure S7 Membrane images of Western blotting

Fig.S4B

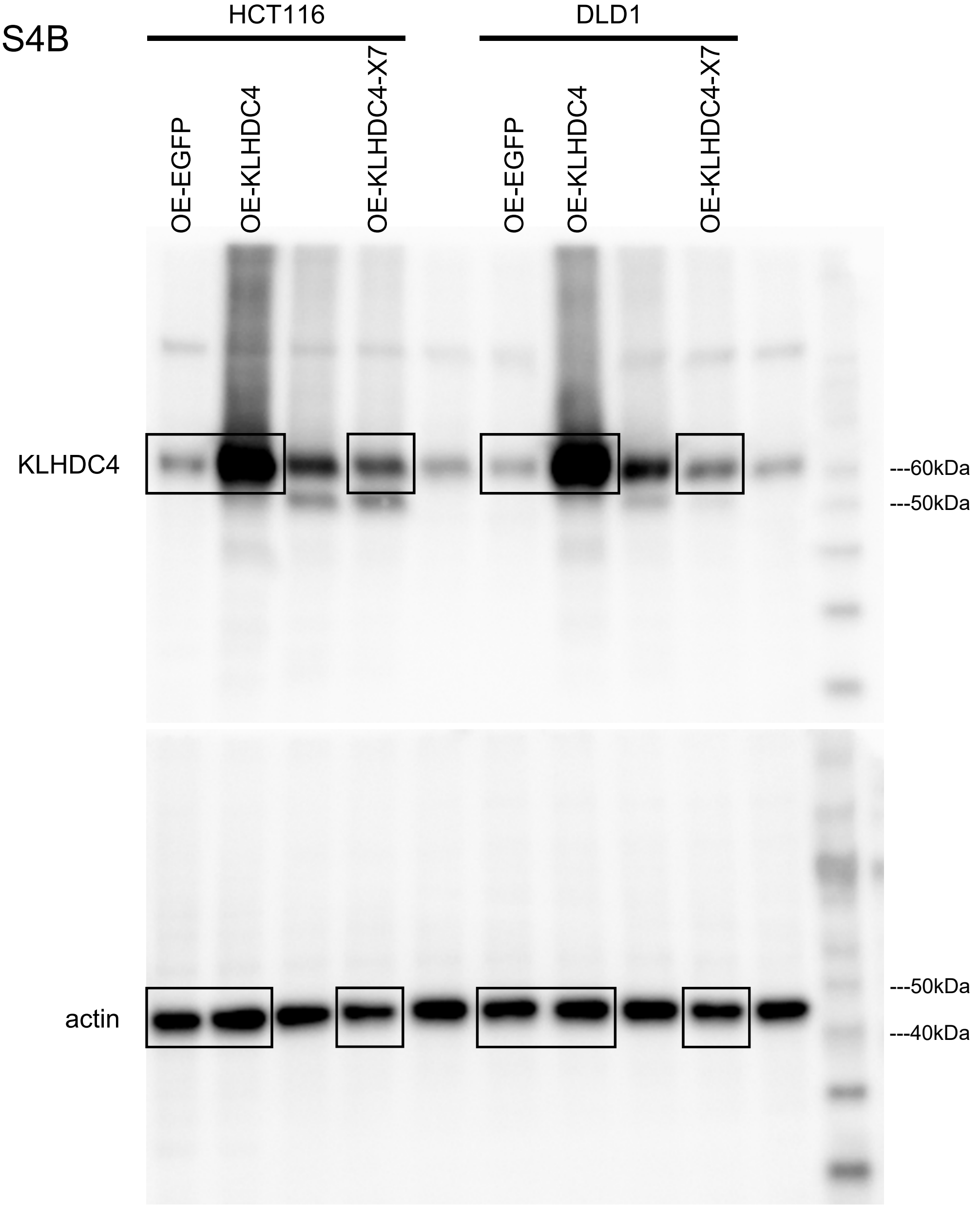

Fig.S5A

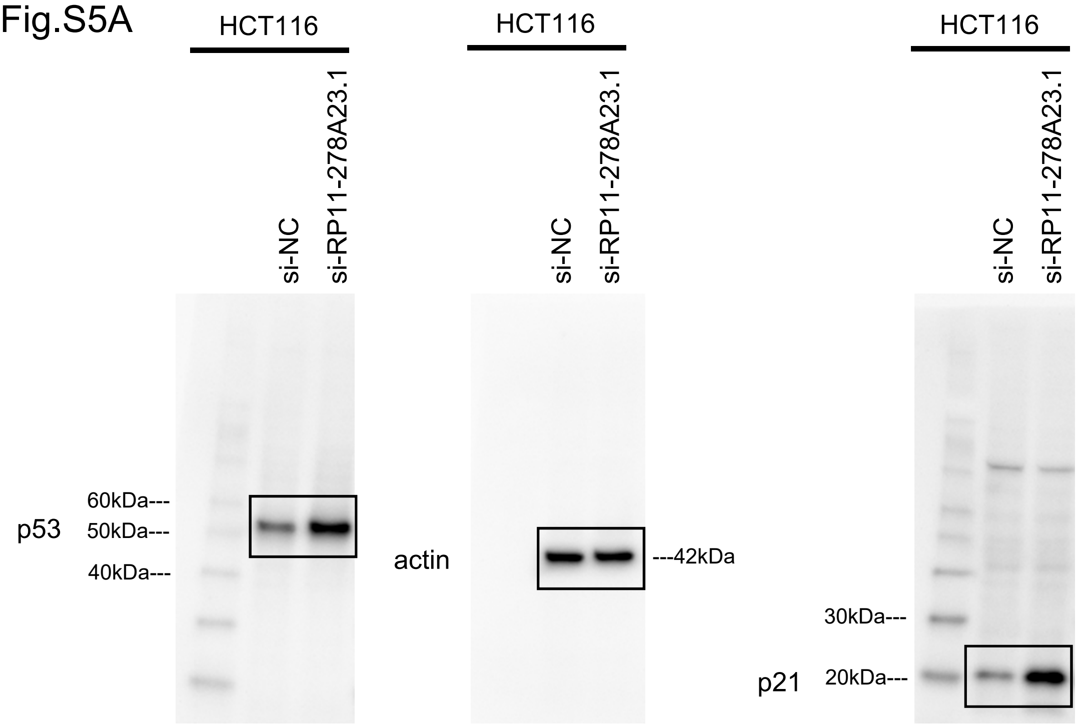

Figure S8 Membrane images of Western blotting in supplementry figures
